# Impact of Secondhand Smoke and E-Cigarette Exposure on Placental Apoptotic and Growth-Regulatory Proteins in Mouse Pregnancy

**DOI:** 10.3390/cells14060453

**Published:** 2025-03-19

**Authors:** Logan Beck, Madison N. Kirkham, Marley Shin, Benjamin T. Bikman, Paul R. Reynolds, Juan A. Arroyo

**Affiliations:** Department of Cell Biology and Physiology, Brigham Young University, Provo, UT 84602, USA

**Keywords:** placenta, apoptosis, secondhand smoke, e-cigarettes, pregnancy

## Abstract

Apoptosis is critical in placental development, and its dysregulation is linked to pregnancy complications such as intrauterine growth restriction (IUGR) and preeclampsia (PE). Environmental exposures, particularly secondhand smoke (SHS) and e-cigarettes (eCigs), may contribute to placental dysfunction through apoptotic pathways. This study examined the effects of SHS and eCig exposure on placental apoptosis and growth-regulatory proteins in a murine model. C57BL/6 pregnant mice were exposed to SHS or eCigs at two critical gestational time points: early trophoblast invasion (E12.5 to E18.5) and established invasion (E14.5 to E18.5). Placental tissues were collected and analyzed for pro-apoptotic and anti-apoptotic markers, heat shock proteins, insulin-like growth factor-binding proteins (IGFBPs), and growth regulators. SHS exposure increased pro-apoptotic markers (BAD, Fas/FasL) and decreased mitochondrial function markers (cytochrome c), indicating compromised cellular survival. Both SHS and eCig exposure reduced anti-apoptotic markers (BCL-2, HSP27, survivin) and growth regulators (IGF-1, IGFBPs). SHS and eCig exposure create a pro-apoptotic environment in the placenta, potentially impairing fetal development through altered apoptotic and growth-regulatory pathways. These findings underscore the risks of environmental exposures during pregnancy, highlighting the need for strategies to minimize maternal exposure to SHS and eCigs.

## 1. Introduction

The placenta is a vital organ for fetal development, facilitating nutrient and oxygen exchange while also regulating immune tolerance and growth signaling. Apoptosis, or programmed cell death, is essential for proper placental development, ensuring trophoblast turnover and vascular remodeling. However, dysregulated apoptosis has been implicated in pregnancy complications such as intrauterine growth restriction (IUGR) and preeclampsia (PE), both of which adversely affect maternal and fetal health [1]. Placental dysfunction in these conditions often involves an imbalance between pro-apoptotic and anti-apoptotic pathways, leading to impaired trophoblast invasion and placental vascularization [2]. Apoptotic regulation in the placenta involves a complex interplay between pro-apoptotic factors, such as BAD and cytochrome c, and anti-apoptotic proteins like BCL-2 and heat shock proteins (HSPs). Disruption of this balance can lead to trophoblast dysfunction, compromising placental health [3]. Additionally, the insulin-like growth factor (IGF) system, modulated by insulin-like growth factor-binding proteins (IGFBPs), plays a critical role in placental growth and nutrient exchange. Dysregulation of IGFBPs has been implicated in both IUGR and preeclampsia, further linking placental apoptosis to adverse pregnancy outcomes [4]. 

Environmental exposures, such as secondhand smoke (SHS) and e-cigarettes (eCigs), represent significant yet modifiable risks to placental and fetal health. SHS exposure has been linked to increased oxidative stress and inflammation, both of which can exacerbate apoptosis and impair placental function [5]. E-cigarettes, while often marketed as safer alternatives, still contain harmful substances, such as nicotine, which has been shown to disrupt placental development and induce apoptosis [6]. Animal studies have shown that nicotine exposure can disrupt trophoblast function and induce apoptosis in placental cells, suggesting that e-cigarettes may pose similar risks to the developing fetus. Prior research in our laboratory demonstrated that SHS and eCigs induce distinct patterns of placental damage, with both exposures creating a pro-apoptotic environment characterized by increased oxidative stress and disrupted growth signaling pathways [7].

This study aims to investigate the effects of SHS and eCig exposure on placental apoptosis and growth-regulatory proteins during rodent pregnancy. We examined the expression of key pro-apoptotic and anti-apoptotic markers, as well as IGFBPs and cell cycle regulators, at two important times in pregnancy (before the start of trophoblast invasion (6 days; starting at 12 days of gestation) and shortly after invasion has already started (4 days; starting at 14.5 days of gestation)) to assess how these exposures might disrupt placental development and function. By elucidating the molecular pathways affected by SHS and eCigs, this research seeks to highlight the potential risks that these exposures pose to pregnancy outcomes and inform strategies to protect maternal and fetal health.

## 2. Methods

### 2.1. Animal and Tissue Preparation

Animal handling and tissue preparation were conducted following previously established protocols in our laboratory [7]. Briefly, C57BL/6 pregnant mice were exposed to SHS or eCigs using a nose-only InExpose smoking system (Scireq, Montreal, QC, Canada) at two critical gestational stages: from embryonic day 12.5 (E12.5) to E18.5 and from E14.5 to E18.5. SHS exposure involved controlled puffs generated from 3R4F research cigarettes (Kentucky Tobacco Research and Development Center, University of Kentucky, Lexington, KY, USA). E-cigarette exposure consisted of vaporized apple-flavored e-liquid containing 6 mg of nicotine delivered at a rate of one puff per minute in an 80 ml airflow. Animals were categorized into five experimental groups (*n* = 6 per group): control (wild-type, room air), SHS exposure (6 or 4 days), and eCig exposure (6 or 4 days). At E18.5, necropsies were performed, and placentas were flash-frozen in liquid nitrogen for protein analysis. The Institutional Animal Care and Use Committee (IACUC) at Brigham Young University approved all procedures.

### 2.2. Apoptotic Molecular Analyses

Placental apoptotic markers were analyzed using a mouse apoptosis antibody array C1 (RayBiotech, Norcross, GA, USA), as previously described [7]. Briefly, 125 μg of total protein lysate per sample was pooled from three animals per group to create two independent sample pools (500 μg/mL each). Membranes were incubated overnight with biotinylated antibodies, followed by detection with a streptavidin-conjugated fluorescent label (Thermo Fisher Scientific, Waltham, MA, USA). Imaging and quantification were performed using the Odyssey DLx Near-Infrared Fluorescence Imaging System (LI-COR, Lincoln, NE, USA) and Image J software (Version 1.54p; U.S. National Institutes of Health, Bethesda, MD, USA).

### 2.3. Statistical Analysis

Data were presented as means ± SE. One-way ANOVA was used to compare protein expression levels among treatment groups. A *p*-value of <0.05 was considered statistically significant. Statistical analysis was conducted using GraphPad Prism 8.0 software.

## 3. Results

### 3.1. Pro-Apoptotic Markers (BAD, Cytochrome c, and FAS/FASL)

**Figure 1:**
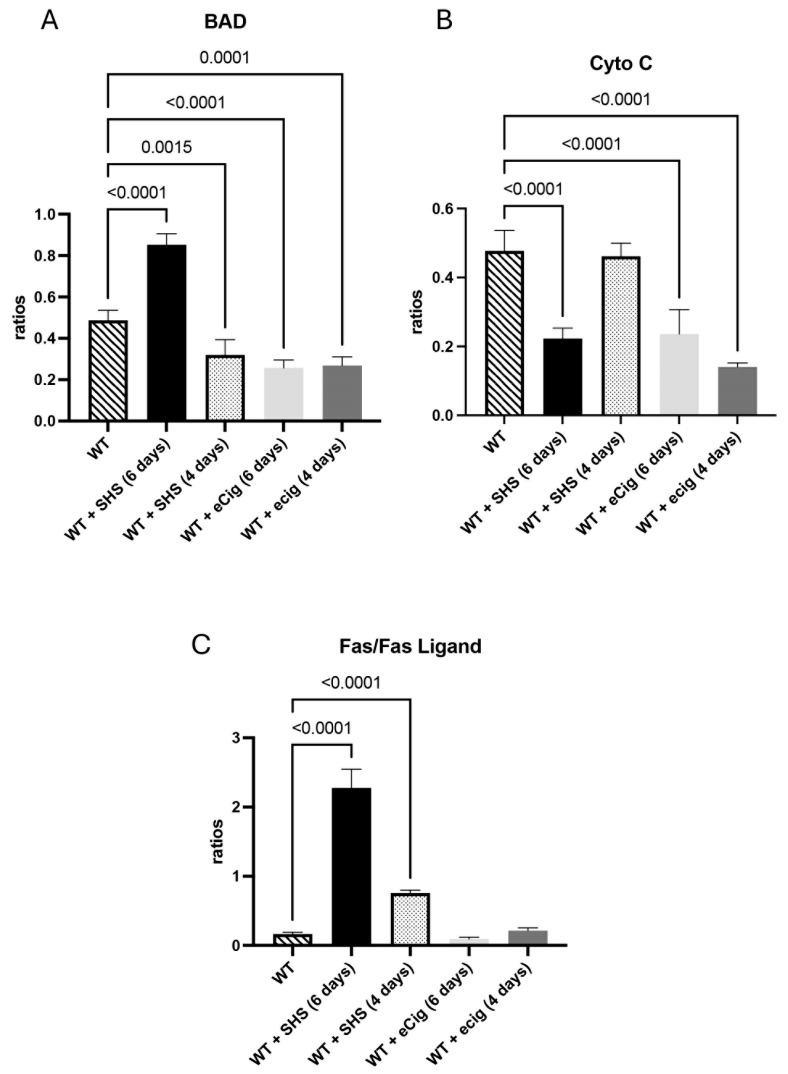
Pro-apoptotic markers BAD, cytochrome c, and Fas/FasL during SHS or eCig treatment. Pro-apoptotic mediators were determined in treated animals, and the results were compared to the controls. Protein expression levels of BAD (**A**), Cyto C (**B**), and (**C**) FAS/FASL were differently regulated by the length and type of treatment compared to the controls. Significant differences are noted as *p* ≤ 0.05.

The BAD protein is known to regulate apoptosis in the placenta [3]. The BAD protein was increased (1.7-fold; *p* < 0.0001) after 6 days of SHS treatment, while a decrease in placental BAD (1.5-fold; *p* < 0.002) was detected in the SHS animals treated for 4 days (Figure 1A). eCig treatment for 6 days showed a decrease in placental BAD protein (1.9-fold; *p* < 0.00) in the treated animals compared to the controls (Figure 1A). The animals treated for 4 days with eCig showed increased BAD protein (1.8-fold; *p* < 0.03) compared to the controls (Figure 1A). Cytochrome c release supports normal placentation and function [8]. We detected a decrease in cytochrome c protein when the animals were exposed to SHS for 6 days (2.1-fold; *p* < 0.0001). The placental cytochrome c protein level was decreased in the eCig animals treated for 6 (2.0-fold; *p* < 0.001) and 4 days (3.4-fold; *p* < 0.001) compared to the controls (Figure 1B). The FAS/FASL protein plays a role in placental development, immune tolerance, and adaption [9]. SHS increased FAS/FASL in the animals treated for 6 days (13.6-fold; *p* < 0.0001) and 4 days (4.6-fold; *p* < 0.000) compared to the controls, while eCigs had no effect (Figure 1C).

### 3.2. Anti-Apoptotic Markers (BCL-2, cIAP-2, HSP27, HSP70, Survivin, and XIAP)

**Figure 2:**
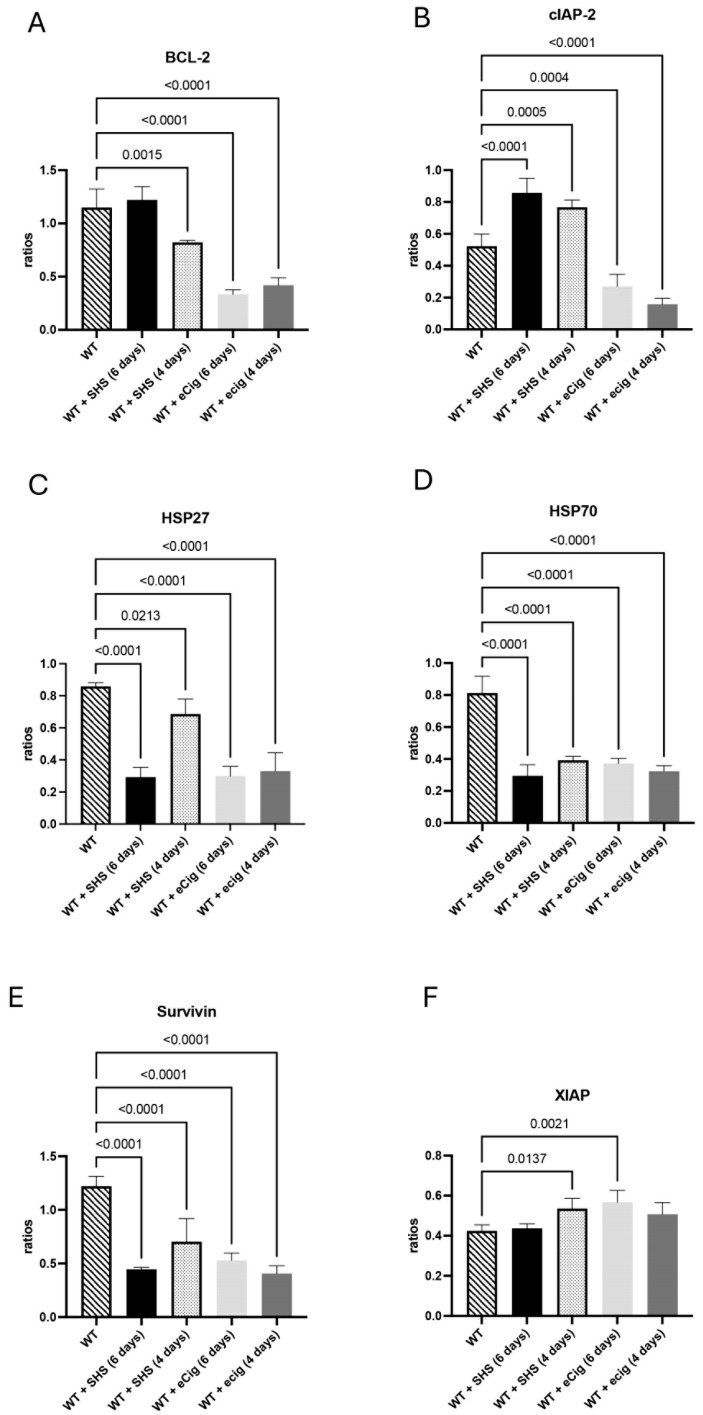
Anti-apoptotic markers BCL-2, cIAP-2, HSP27, HSP70, Survivin, and XIAP during SHS or eCig treatment. Anti-apoptotic mediators were determined in the treated animals, and the results were compared to the controls. Protein expression levels of BCL-2 (**A**), cIAP-2 (**B**), HSP27 (**C**), HSP70 (**D**), Survivin (**E**), and XIAP (**F**) were differently regulated by the length and type of treatment when compared to the controls. Significant differences are noted as *p* ≤ 0.05.

BCL-2 supports placenta cells’ survival during stress [10]. The BCL-2 protein was decreased in the placenta of the animals treated with SHS for 4 days (1.4-fold; *p* < 0.002; Figure 2A). Similarly, BCL-2 was also reduced in the placenta of the animals treated with eCigs for both 6 (3.4-fold; *p* < 0.0001) and 4 days (2.7-fold; *p* < 0.0001; Figure 2A). cIAP-2 inhibits apoptosis and regulates inflammation [11]. The cIAP-2 protein was increased in the placenta of the animals treated with SHS for 6 days (1.6-fold; *p* < 0.0001) and 4 days (1.5-fold; *p* < 0.0006; Figure 2B). In contrast, a decrease in placental cIAP-2 was detected in the placenta of the animals treated with eCigs for 6 (1.9-fold; *p* < 0.0005) and 4 days (3.3-fold; *p* < 0.0001; Figure 2B). HSP27 is a small heat shock protein involved in apoptotic regulation [12]. HSP27 was decreased in the placenta of the animals treated with SHS or eCigs at all the exposure times studied (average of 2.4; *p* < 0.03; Figure 2C). HSP70 is a heat shock proteins that inhibit apoptotic pathways [13]. We detected a decrease in HSP70 (2.4-fold; *p* < 0.0001) in the placenta of the animals treated with SHS or eCigs at all the exposures times studied (Figure 2D). Survivin promotes trophoblast cell survival, supporting placental development and function [14]. Treatment with SHS for 6 or 4 days decreased placental survivin compared to the controls (average of 1.7-fold; *p* < 0.000; Figure 2E). Similarly, survivin was also reduced when the animals were treated for 6 and 4 days with eCigs (average 2.7-fold; *p* < 0.0001; Figure 2E). XIAP is a member of the inhibitor of the apoptosis protein (IAP) family that inhibits caspases to prevent apoptosis in placental cells [15]. An increase in XIAP protein (1.3-fold, *p* < 0.02) was observed in the placenta of the animals treated with SHS for 4 days (Figure 2F). This increase in XIAP protein was also observed (1.2-fold; *p*< 0.003) when the animals were treated with eCigs for 6 days (Figure 2F).

### 3.3. IGFBP Family and Growth Regulation

**Figure 3:**
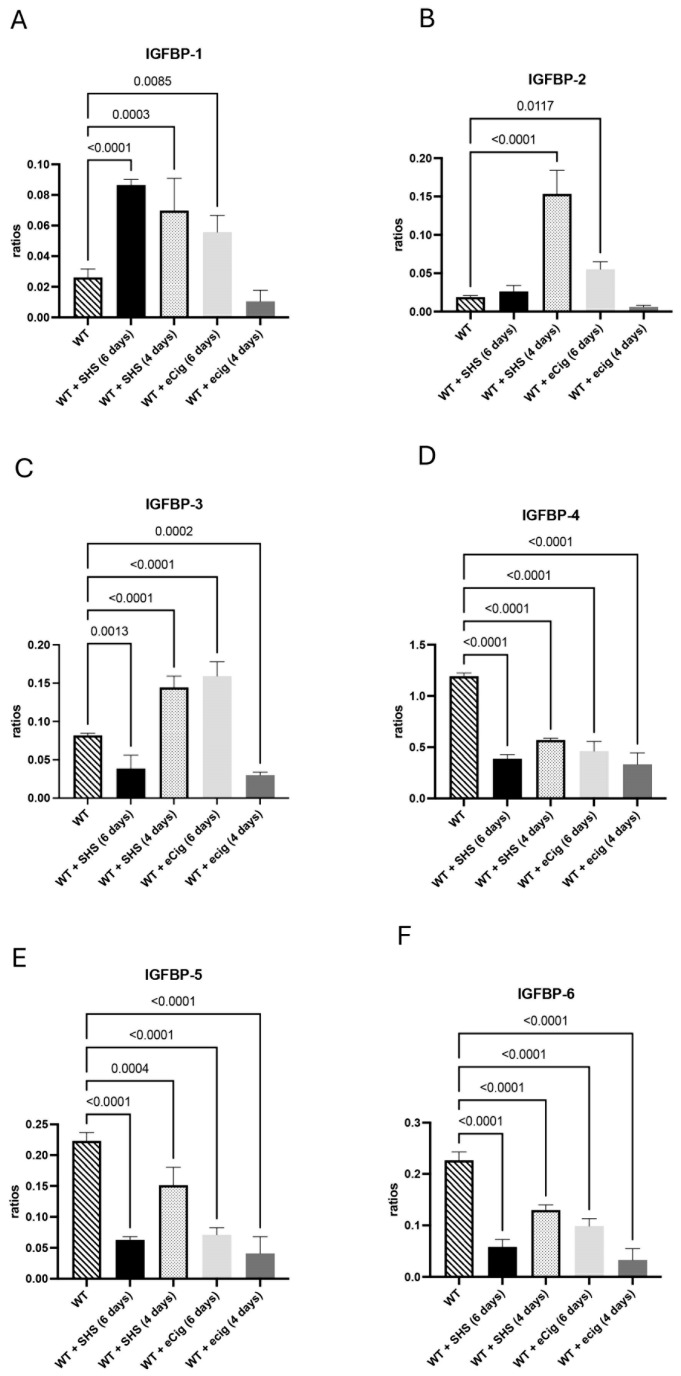
IGFBP growth regulation markers IGFBP-1, IGFBP-2, IGFBP-3, IGFBP-4, IGFBP-5, and IGFBP-6 during SHS or eCig treatment. IGFBP growth regulation mediators were determined in the treated animals compared to the controls. Protein expression levels of IGFBP-1 (**A**), IGFBP-2 (**B**), IGFBP-3 (**C**), IGFBP-4 (**D**), IGFBP-5 (**E**), and IGFBP-6 (**F**) were differently regulated by the length and type of treatment when compared with the controls. Significant differences are noted as *p* ≤ 0.05.

The IGFBP family of proteins is produced in multiple tissues and is involved in growth regulation and apoptotic signaling [16]. There was an increase in IGFBP1 in the placenta of animals treated for 6 and 4 days with SHS (both at 3.0-fold; *p* < 0.0004) compared to the controls (Figure 3A). In the animals treated with eCigs, IGFBP-1 was only increased (2.1-fold; *p* < 0.009) within 6 days of treatment (Figure 3A). SHS increased the IFGBP-2 protein levels within 4 days of treatment (8.1-fold; *p* < 0.001) compared to the controls (Figure 3B). The eCig treatment increased placental IGFBP-2 within 6 days of treatment (2.9-fold; *p* < 0.02) in the treated animals compared to the controls (Figure 3B). We detected decreased IGFBP-3 (2.1-fold; *p* < 0.002) in the placentas of the animals treated with SHS for 6 days. In contrast, 4 days of SHS treatment increased placental IGFBP-3 protein (1.8-fold; *p* < 0.0001; Figure 3C). In contrast, we observed that placental IGFBP-3 was increased with 6 days of eCig treatment, while a decrease was detected in the animals treated for 4 days with eCigs (Figure 3C). In the animals treated for 6 or 4 days with either SHS or eCigs, we detected a decrease in IGFBP4 (average of 2.8-fold; *p* < 0.0001;), IGFBP-5 (average of 3.4-fold; *p* < 0.0005), and IGFBP6 (average of 3.7-fold; *p* < 0.0001) compared to the controls (Figure 3D–F).

### 3.4. Cell Cycle and Growth Inhibition Markers (p27 and IGF-1)

**Figure 4:**
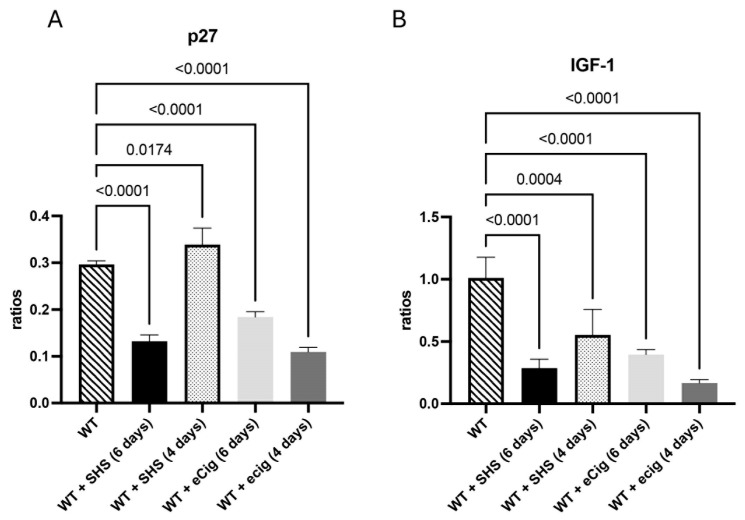
Cell cycle and growth inhibition markers p27 and IGF-1 during SHS or eCig treatment. Cell cycle and growth inhibition mediators were determined in the treated animals, and the results were compared to the controls. Protein expression levels of p271 (**A**) and IGF-1 (**B**) were differently regulated by the length and type of treatment when compared to the controls. Significant differences are noted as *p* ≤ 0.05.

p27 plays a role in modulating cell turnover, apoptosis, and maintaining placental structure [17]. The animals treated for 6 days with SHS had decreased p27 (2.3-fold; *p* < 0.0001), while SHS treatment for 4 days increased p27 levels (1.2-fold; *p* < 0.02) in the placenta of the treated animals compared to the controls (Figure 4A). eCig treatment for 6 or 4 days decreased placental p27 protein (average of 2.1-fold; *p* < 0.0001) compared to the controls (Figure 4A). IGF-1 is pivotal for promoting placental cell proliferation, inhibiting apoptosis, and supporting overall placental function [18]. Placental IGF-1 was decreased when the animals were treated for 6 or 4 days with either SHS or eCigs compared to the controls (average of 4.0-fold; *p* < 0.0005; Figure 4B).

## 4. Discussion

This study demonstrates that SHS and eCig exposure alters placental apoptotic and growth-regulatory pathways, potentially impairing fetal development. Increased pro-apoptotic marker expression and reduced mitochondrial function suggest oxidative stress as a key mechanism underlying these changes. The downregulation of IGFBP and IGF-1 further indicates disruptions in placental growth regulation.

The findings reinforce concerns regarding environmental exposure during pregnancy, particularly the potential risks associated with e-cigarette use. While SHS exposure activates immune-related apoptotic pathways, eCigs may primarily disrupt mitochondrial function. These results suggest that e-cigarettes are not a safe alternative to traditional smoking during pregnancy.

### 4.1. Pro-Apoptotic Markers

This study found significant alterations in the expression of pro-apoptotic proteins in response to both SHS and eCig exposure. Increased levels of the pro-apoptotic marker BAD after prolonged SHS exposure align with previous findings suggesting that environmental toxins can induce apoptosis in placental cells, potentially compromising placental health and function [3]. BAD expression, however, decreased with shorter SHS exposure, suggesting a nuanced response to stress duration. Interestingly, eCig exposure produced an opposite pattern in BAD expression between short- and long-term exposure, indicating that different mechanisms might be at play with these two environmental exposures.

Cytochrome c, a key mediator of the intrinsic apoptosis pathway, was significantly reduced in response to SHS and eCig exposure. This reduction implies that mitochondrial function is compromised, which could impair trophoblastic cells’ ability to meet the growing fetus’s metabolic demands [19]. Given that cytochrome c release is often stimulated by oxidative stress, a known consequence of tobacco smoke exposure, these findings are consistent with oxidative injury as a potential mechanism underlying the observed placental dysfunction [20].

The Fas/FasL pathway, central to immune regulation and extrinsic apoptosis, was upregulated in response to SHS exposure. This pathway has been implicated in the pathophysiology of preeclampsia, where increased placental cell turnover due to apoptosis is often observed [21]. ECig exposure did not affect Fas/FasL levels, suggesting that SHS may uniquely activate immune-related apoptotic pathways, potentially due to the specific toxins and inflammatory mediators in conventional tobacco smoke.

### 4.2. Anti-Apoptotic Markers

SHS and eCig exposure was associated with decreased levels of the anti-apoptotic protein BCL-2, suggesting a shift towards apoptosis in the placental cells. This decrease in BCL-2 is consistent with findings in placentas affected by preeclampsia, where a pro-apoptotic environment may contribute to impaired placental function and adverse fetal outcomes [22]. Heat shock proteins, including HSP27, HSP60, and HSP70, were downregulated across all exposure conditions. Heat shock proteins are crucial for cellular stress response and survival, and their reduction suggests that the placenta’s capacity to handle oxidative or inflammatory stress may be compromised [23].

Survivin, a protein which inhibits apoptosis and supports cell survival, was significantly reduced following SHS and eCig exposure. Survivin is essential in placental development, particularly in maintaining trophoblast cell viability. The reduction in survivin expression is consistent with a pro-apoptotic environment, which could impair placental function and contribute to conditions such as IUGR [14].

### 4.3. IGFBP Family and Growth Regulation

IGFBPs are critical regulators of fetal growth, and their dysregulation could profoundly affect fetal development. The increased levels of IGFBP-1 and IGFBP-2 with SHS exposure suggest an adaptive response to nutrient restriction, as these proteins often increase under conditions of fetal stress to modulate the activity of insulin-like growth factors [4]. The differential expression patterns of IGFBP-3 in response to SHS and eCig exposure point to complex regulatory mechanisms that the specific type of environmental exposure may influence. Depending on the context, IGFBP-3’s dual role in promoting and inhibiting apoptosis aligns with its varied response observed in this study [24].

Reductions in IGFBP-4, IGFBP-5, and IGFBP-6 in both the SHS and eCig exposure groups indicated potential disruptions in IGF signaling, which is crucial for trophoblast migration and placental development. These reductions could impact nutrient transport and cell survival, essential for healthy fetal growth [25].

### 4.4. Cell Cycle and Growth Inhibition Markers

The cell cycle inhibitor p27 showed a complex expression pattern, with decreased levels following prolonged SHS exposure and increased levels with shorter SHS exposure. This finding may reflect an adaptive response aimed at modulating cell turnover under stress conditions [26]. However, the consistent decrease in p27 with eCig exposure suggests that eCigs might induce a more sustained impairment of cell cycle regulation in the placenta.

IGF-1, a key growth factor promoting cell proliferation and survival, was consistently downregulated in all exposure groups. Reduced IGF-1 levels could lead to decreased placental growth and nutrient transfer capacity, crucial for fetal development. This finding is consistent with reports of reduced IGF-1 levels in placentas affected by IUGR [24].

## 5. Conclusions

In summary, the findings suggest that SHS and eCig exposure create a pro-apoptotic environment in the placenta, compromising cellular survival and growth regulation through mechanisms involving intrinsic (mitochondrial) and extrinsic (immune-mediated) apoptotic pathways. These changes could compromise placental function, affecting nutrient and oxygen transport and ultimately impacting fetal growth. IGFBP and IGF signaling alterations suggest that SHS and eCig exposure may impair growth-regulatory pathways essential for fetal development.

This study highlights the potential risks associated with eCig exposure during pregnancy, as well as the well-documented dangers of SHS, reinforcing the need for public health strategies to minimize pregnant individuals’ exposure to these environmental toxins. Further studies are warranted to delineate the precise molecular mechanisms by which SHS and eCig exposure affect placental function and evaluate whether similar effects occur in human pregnancies.

## Data Availability

The original contributions presented in this study are included in the article; further inquiries can be directed to the corresponding author.
